# Acupuncture Delays Cartilage Degeneration through Upregulating SIRT1 Expression in Rats with Osteoarthritis

**DOI:** 10.1155/2021/2470182

**Published:** 2021-12-31

**Authors:** Hui Liu, Tingting Zhang, Min Liu, Chunhong Wang, Jinfeng Yan

**Affiliations:** ^1^Department of Traditional Chinese Medicine, The Affiliated Hospital of Qingdao University, Qingdao, China; ^2^Department of Rehabilitation Medicine, The Affiliated Hospital of Qingdao University, Qingdao, China

## Abstract

Silent mating type information regulation 2 homolog 1 (SIRT1) has been reported to inhibit osteoarthritic gene expression in chondrocytes. Here, efforts in this study were made to unveil the specific role of SIRT1 in the therapy of acupuncture on cartilage degeneration in osteoarthritis (OA). Specifically, OA was established by the anterior cruciate ligament transection method in the right knee joint of rats, subsequent to which acupuncture was performed on two acupoints. Injection with shSIRT1 sequence–inserted lentiviruses was conducted to investigate the role of SIRT1 in acupuncture-mediated OA. Morphological changes and cell apoptosis in rat OA cartilages were examined by safranin-O staining and terminal deoxynucleotidyl transferase-mediated nick-end labeling (TUNEL) assay, respectively. The serum levels of tumor necrosis factor (TNF)-*α* and interleukin (IL)-2 in OA rats were assessed by enzyme-linked immunosorbent assay (ELISA). The expressions of SIRT1, cartilage matrix degradation-related proteins (matrix metalloproteinase (MMP)-9 and ADAMTS5), NF-*κ*B signaling-related markers (p-p65/p65 and p-I*κ*B*α*/I*κ*B*α*), and cartilage matrix synthesis-related proteins (collagen II and aggrecan) in the OA cartilage were analyzed by western blot. As a result, acupuncture counteracted OA-associated upregulation of TNF-*α*, IL-2, cartilage matrix degradation-related proteins, and NF-*κ*B signaling-related markers, morphological damage, apoptosis, SIRT1 downregulation, and loss of cartilage matrix synthesis-related proteins in rat articular cartilages. SIRT1 silencing reversed acupuncture-induced counteractive effects on the aforementioned OA-associated phenomena (except apoptosis, the experiment regarding which under SIRT1 silencing was not performed). Collectively, acupuncture inhibited chondrocyte apoptosis, inflammation, NF-*κ*B signaling activation, and cartilage matrix degradation by upregulating SIRT1 expression to delay OA-associated cartilage degeneration.

## 1. Introduction

Osteoarthritis (OA) is a chronic, incurable, and destructive joint disease characterized by chondrocyte hypertrophy and cartilage degeneration [1]. Cartilage degeneration, which is caused by mechanical stresses, inflammation, or aging, results in friction between bones, further leading to the occurrence of OA [2, 3]. Extracellular matrix (ECM), a complex network within cartilages, surrounds chondrocytes which preserve the structural and functional integrity of the articular cartilages by synthesizing ECM [3, 4]. Elevated inflammatory responses in cartilages can aggravate ECM degradation and restrain cartilage intrinsic repair, thus destroying cartilage homeostasis to eventually induce osteoarticular diseases such as OA [3, 5, 6]. Besides, as the only cell type present in the cartilage, chondrocytes can play a causative role for OA when its apoptosis is dysregulated [7]. Multiple treatment methods for OA have been performed, including oral intake of nonsteroidal anti-inflammatory drugs (NSAIDs) and glucosamine, which could only achieve the goal of pain relief; however, they are insufficient to prevent or cure patients with OA [8]. Moreover, surgery, such as arthroscopic partial meniscectomy which remains the last resort for the patients, exhibits limited therapeutic effects, with randomized clinical trials showing its benefits for knee function and pain persisting for only 1-2 years [9, 10]. Therefore, strategies with long-lasting effect against inflammatory responses, ECM degradation, and apoptosis are necessary for modulating cartilage degeneration and thus alleviating OA pathology.

Acupuncture, which is applied as a complementary and alternative therapy in Western countries, has gained increasing acceptance by virtue of its safe, cost-effective, and long-lasting effects, as demonstrated by pain reduction and alleviation of some disease-specific symptoms on various diseases such as functional dyspepsia [11], psoriasis [12], and chronic prostatitis/chronic pelvic pain syndrome [13]. Several systematic reviews regarding the effect of acupuncture in the treatment of OA have indicated that acupuncture can reduce pain and improve functional activity for patients with OA in the knee [14, 15] and in the hip [16]. However, some reports have manifested that pain relief and other functions of acupuncture are likely attributed to patients' greater expectations for these effects [17, 18]. Hence, there is uncertainty about the underlying mechanism of acupuncture in the treatment of OA.

A recent study has proven that acupuncture can target the silent mating type information regulation 2 homolog 1 (SIRT1) in the hypothalamic arcuate nucleus to perform an anorectic effect, thereby improving obesity in high-fat-diet-induced rats with insulin resistance [19]. SIRT1 is a deacetylase that can promote mammalian cell survival through a mechanism where it deacetylates the DNA repair factor Ku70, leading to sequestration of the proapoptotic factor Bax and thus inhibiting stress-induced cell apoptosis [20]. Overexpression of SIRT1 is found to inhibit interleukin (IL)-1*β*-induced expressions of cartilage-degrading enzymes in human chondrocytes [21]. Therefore, this study was designed to delve into the specific role of SIRT1 in the mechanism underlying acupuncture-induced regulation on OA pathology.

## 2. Material and Methods

### 2.1. Ethics Statement

All animal experiments were performed in The Affiliated Hospital of Qingdao University according to the guidelines of the China Council on Animal Care and Use. This study was approved by the Committee of Experimental Animals of The Affiliated Hospital of Qingdao University (approval number: DO202006016). Every effort was made to minimize pain and discomfort to the animals.

### 2.2. Construction of shSIRT1 Lentiviruses

ShSIRT1 oligonucleotide sequences were designed and processed into shSIRT1 lentivirus vectors by GeneChem Co., Ltd (Shanghai, China). Briefly, the shSIRT1 oligonucleotide sequences were inserted into the lentivirus expression vector pGCSIL (GeneChem Co., Ltd.) containing a CMV-driven GFP reporter, after double enzyme digestion at two restriction endonuclease sites *Asc*I and *Pmel* was conducted. Scrambled shRNA sequences were used as the negative control (shNC). Then, the recombinant vector and the packaging vectors pHelper 1.0 and 2.0 (GeneChem Co., Ltd.) were cotransfected into 293T cells by lipofectamine 3000 reagent (L3000015, ThermoFisher, Waltham, MA, USA) to produce viral particles of shSIRT1 and shNC.

### 2.3. Establishment of OA Rats and shSIRT1-Lentivirus Injection

Male Sprague–Dawley rats (*n* = 70), weighing 180 to 220 g and aged 8 weeks old, were purchased from SLAC (Shanghai, China). The rats were housed under a specific condition, namely, 25 ± 1°C, 50∼55% humidity, and a 12 h light/12 h dark circadian cycle, with free access to regular chow diet. After being habituated to the environment for one week, all rats were randomly divided into seven groups (*n* = 10): Sham group, Model group, Model + Acupuncture group, Model + shNC group, Model + shSIRT1 group, Model + Acupuncture + shNC group, and Model + Acupuncture + shSIRT1 group. The anterior cruciate ligament transection (ACLT) method was used to induce OA [22] in Model-related groups. Under anesthesia using 2% pentobarbital sodium (P-010, 0.35 g Sigma-Aldrich, St. Louis., MO, USA), the rats suffered from a 2 cm long incision in the right knee joint. After the medial ligament was exposed in the opened joint capsule, a lateral dislocation of the knee was conducted. Then, the anterior cruciate ligament was cut off, and cefotiam hydrochloride (1.0–3.0 mg, 1098005, Sigma-Aldrich, USA) was used to treat the incision for preventing postoperative infection. For the Sham group, rats did not receive transection of the anterior cruciate ligament. After OA induction, rats in Model + shSIRT1 group were intra-articularly injected with 1 × 10^9^ plaque-forming units of lentivirus vectors with shSIRT1 sequences or scrambled shRNA sequences that had diluted in 100 *µ*L phosphate buffered saline (PBS; P5493, Sigma-Aldrich, USA). At the 8^th^ week after the operation, all the rats were sacrificed via spinal dislocation. Rat blood was collected and cartilaginous tissues were excised from the right knee joints of the rats.

### 2.4. Acupuncture Treatment

In the acupuncture-related groups, acupuncture was performed on the acupoint Dubi which is located in the depression lateral to the patellar ligament and on the acupoint Zusanli which is located 3 cun lower than the Dubi (16 cun equal to the distance between the knee joint and the external ankle) [22]. These two acupoints were inserted by needles (0.25 mm diameter ×40 mm length), followed by 1 min manual twisting of the needles with a frequency of 2 Hz. Then, the needles remained still for 4 min. The whole operating procedure was repeated six times.

### 2.5. Safranin-O Staining

Cartilaginous tissues were fixed in 4% paraformaldehyde, dehydrated by gradient ethanol (1.00983, Sigma-Aldrich, USA), transparentized by xylene (95682, Sigma-Aldrich, USA), and subsequently embedded in paraffin (1496904, Sigma-Aldrich, USA). After being sliced into 5 *μ*m thick by using a slicer (pfm3005 E, Dakewe Biotech Co., Ltd, Shenzhen, China), the tissues were dewaxed by using xylene and rehydrated by using gradient ethanol. Later, hematoxylin solution (H3136, Sigma-Aldrich, USA) was used to stain the tissues for 5 min. After being quickly destained through 2–3 dips of acid EtOH (56694, Sigma-Aldrich, USA) and then washed in running water for 1 min, the tissues were stained with fast green solution (210-M, Sigma-Aldrich, USA) for 5 min, rinsed in 1% acetic acid solution (A6283, Sigma-Aldrich, USA) for 10 sec, and eventually stained with safranin-O solution (S2255, Sigma-Aldrich, USA) for 5 min. The stained tissues were dehydrated by gradient ethanol, transparentized by xylene, and mounted with Canada balsam (60610, Sigma-Aldrich, USA). An inverted microscope (IX71; Olympus, Tokyo, Japan) was used to observe the cartilaginous tissues under ×100 magnification, following which the Osteoarthritis Research Society International (OARSI) scoring system was used to quantify cartilage degenerative degree, as previously described [23]. The scoring scale ranged from 0 to 6, with 0 for surface intact and cartilage morphology intact, 1 for surface intact, 2 for surface discontinuity, 3 for vertical fissures (clefts), 4 for erosion, 5 for denudation, and 6 for deformation. The OARSI scoring was performed by two independent experienced researchers who were not informed about the objective of the study.

### 2.6. Terminal Deoxynucleotidyl Transferase-Mediated Nick-End Labeling (TUNEL) Assay

A TUNEL assay kit (ab66108, Abcam, Cambridge, MA, USA) was used to assess cell apoptosis in rat cartilaginous tissues. Rat cartilages were chopped into 0.5 mm thick tissues, then washed with PBS containing 10 mL/L penicillin-streptomycin (P4333, Sigma-Aldrich, USA), and digested in 0.25% trypsin (T2600000, Sigma-Aldrich, USA) for 1 h. Subsequently, digestion with type II collagenase was performed at 37°C overnight. When single cells were captured under an inverted microscope (IX71; Olympus, Tokyo, Japan), Dulbecco's modified Eagle medium (DMEM; A4192101, ThermoFisher, USA) was added to stop the digestion, and chondrocytes were harvested after centrifugation at 1000 × *g* for 5 min. Following fixation with 4% paraformaldehyde for 15 min on ice, chondrocytes were washed with PBS, incubated with ice-cold 70% ethanol for 30 min, and separated by 0.1% Triton X-100 (X100, Sigma-Aldrich, USA). Then, apoptotic chondrocytes were colored in staining solution at 37°C for 60 min and subsequently counterstained by 4′,6-diamidino-2-phenylindole (DAPI, D9542, Sigma-Aldrich, USA) for 7 min. The fluorescent chondrocytes were captured by an inverted fluorescence microscope (ECLIPSE Ti, Nikon, Tokyo, Japan) under ×200 magnification.

### 2.7. Enzyme-Linked Immunosorbent Assay (ELISA)

The serum levels of tumor necrosis factor (TNF)-*α* and interleukin (IL)-2 were assessed by ELISA kits (JL13202-96T and JL11302-96T, Jonln Biotechnology, Shanghai, China, http://www.jonln.com/). Briefly, rat serum (50 *μ*L) was centrifugated from the rat blood at 1000×*g* for 20 min, which was added into each well of the enzyme-coated plates. Then, the plates were incubated with horseradish peroxidase-labeled antibodies (100 *μ*L) at 37°C for 60 min. Afterwards, the plates were washed with the buffer solution (350 *μ*L) and stood for 1 min. After being dried by using an absorbent paper, the plate was added with the substrate reagent and incubated at 37°C for 15 min in the dark. The reaction was terminated by adding the stop solution, and the optical density at 450 nm was recorded by using a microplate reader (ELx808, BioTek, Winooski, VT, USA).

### 2.8. Western Blotting

Rat OA cartilages were processed by using a laboratory homogenizer (Shanghai Donghua, Shanghai, China), and total protein from the cartilaginous homogenate was isolated by using RIPA buffer (89900, ThermoFisher, USA). Protein concentration was determined by using BCA kits (A53227, ThermoFisher, USA). The isolated protein (35 *μ*g) and marker (4 *μ*L) (PR1910, Solarbio, Beijing, China) were separately loaded, dispersed by 8% or 10% or 12% SDS-PAGE gel (P0678 or P0670 or P0672, Beyotime, Shanghai, China), and then transferred onto PVDF membranes (P2438, Sigma-Aldrich, USA). The membranes were blocked by 5% nonfat milk (P2194, Sigma-Aldrich, USA) in tris buffered saline with 1% Tween 20 (TBST; TA-125-TT, ThermoFisher, USA) for 1 h and further probed with primary antibodies against SIRT1 (#9475, 120 kDa, 1 : 1000, Cell Signaling Technology, Danvers, MA, USA), matrix metallopeptidase (MMP)-9 (ab76003, 92 kDa, 1 : 1000, Abcam, USA), A disintegrin and metalloproteinase with thrombospondin motifs 5 (ADAMTS5; ab41037, 73 kDa, 1 : 250, Abcam, USA), phosphorylated (p)-p65 (#3033, 62 kDa, 1 : 1000, Cell Signaling Technology, USA), p65 (#8242, 65 kDa, 1 : 1000, Cell Signaling Technology, USA), p-I*κ*B*α* (#2859, 40 kDa, 1 : 1000, Cell Signaling Technology, USA), I*κ*B*α* (#4812, 39 kDa, 1 : 1000, Cell Signaling Technology, USA), collagen II (ab188570, 141 kDa, 1 : 1000, Abcam, USA), aggrecan (orb624552, 250 kDa, 1 : 500, Biorbyt, Cambridge, UK), and GAPDH (ab8245, 36 kDa, 1 : 1000, Abcam, USA) at 4°C overnight. Later, following being washed with TBST, the membranes were incubated with a secondary antibody goat anti-rabbit IgG (A32731, 1 : 10000, ThermoFisher, USA) or goat anti-mouse IgG (A-21058, 1 : 10000, ThermoFisher, USA). Immunoreactive bands were visualized by using an enhanced chemiluminescence reagent kit (WP20005, ThermoFisher, USA) with an imaging device (iBright CL750, ThermoFisher, USA) and analyzed quantitatively by using ImageJ software (1.52s version, National Institutes of Health, Bethesda, MA, USA).

### 2.9. Statistical Analysis

Statistical analyses were performed using GraphPad Prism (version 8.0, GraphPad Software Inc., San Diego, CA, USA). Measurement data were obtained from independent experiments performed in triplicate and were presented as mean ± standard deviation. Differences between two groups were analyzed by the independent *t*-test, and those among multiple groups were analyzed by one-way analysis of variance (ANOVA) followed by Tukey's posthoc test. For analyzing the data of articular cartilage damage (score analyzing), a nonparametric test (Kruskal–Wallis test) was performed. *P* < 0.05 was regarded statistically significant.

## 3. Results

Acupuncture counteracted OA-associated inflammation, morphological damage, and apoptosis in rat articular cartilages.

**Figure 1:**
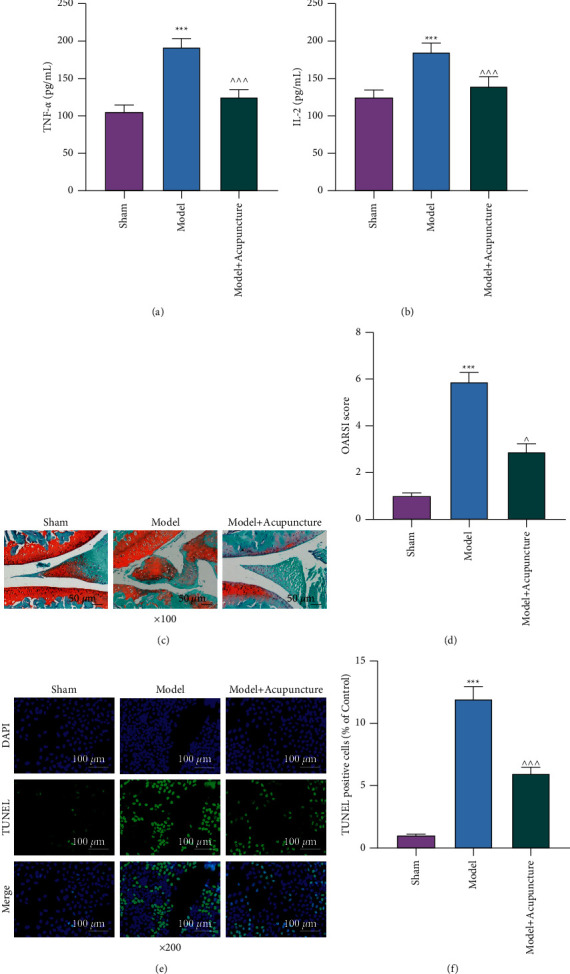
Acupuncture counteracted OA-associated inflammation, morphological damage, and apoptosis in rat articular cartilages. (a/b): The levels of TNF-*α* and IL-2 in the serum of OA rats treated with or without acupuncture were assessed by enzyme-linked immunosorbent assay. (c/d): Morphological damage in the articular cartilage of OA rats treated with or without acupuncture was examined by safranin-O staining (magnification: ×100; scale: 50 *µ*m). (e/f): Apoptosis of the articular cartilage of OA rats treated with or without acupuncture was assessed by the TUNEL assay (magnification: ×200; scale: 100 *µ*m). ^*∗∗∗*^*P* or ^ ^ ^*P* < 0.001; ^*∗*^ vs. Sham; ^ vs. Model (OA: osteoarthritis; TNF-*α*: tumor necrosis factor-*α*; IL-2: interleukin-2; TUNEL: terminal deoxynucleotidyl transferase-mediated nick-end labeling).

After OA was induced in rats, the levels of TNF-*α* and IL-2 in the serum were assessed by ELISA, revealing that these levels were significantly higher in OA rats than in Sham-operated rats (*P* < 0.001; Figure 1(a) and 1(b)). Then, acupuncture was performed, which resulted in reversals in the increment of the two levels in OA rats (*P* < 0.001; Figures 1(a)–1(b)). Meanwhile, 3 safranin-O staining assays indicated that OA rat articular cartilages were notably damaged, resulting in cartilage erosion, proteoglycan, and cell loss in the morphology, when compared to articular cartilages of Sham-operated rats, and the OA cartilages were rated as 6 on the OARSI scoring system (Figures 1(c)–1(d)). Treatment with acupuncture ameliorated the abovementioned morphological damage and regulated the OARSI score to 3 (Figures 1(c)–1(d)). Moreover, the TUNEL assay presented a marked enhancement of chondrocyte apoptosis in OA rat articular cartilages in contrast with Sham-operated rat articular cartilages. Also, treatment with acupuncture reversed the apoptosis enhancement (Figures 1(e)–1(f)).

### 3.1. Acupuncture Reversed OA-Associated Regulation of the Expressions of SIRT1, MMP-9, ADAMTS-5, and the NF-*κ*B Signaling Pathway in Rat Articular Cartilages

**Figure 2:**
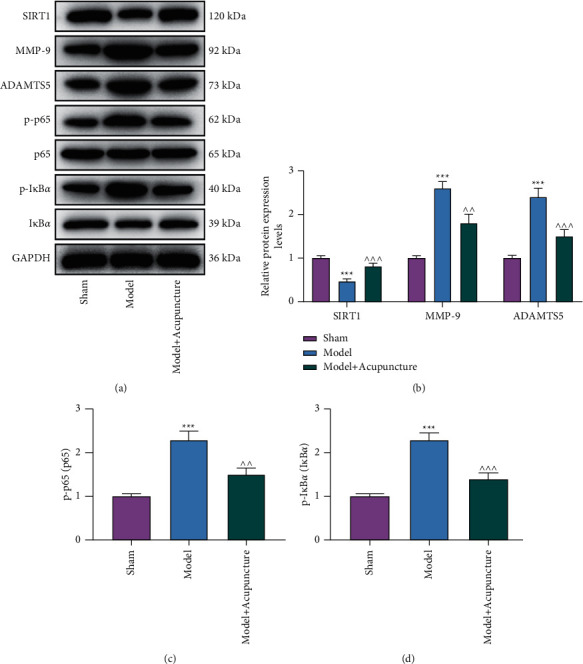
Acupuncture reversed OA-associated regulation of the expressions of SIRT1, MMP-9, ADAMTS-5, and the NF-*κ*B signaling pathway in rat articular cartilages. (a/b/c/d): The expressions of SIRT1, MMP-9, ADAMTS5, p-p65/p65, and p-I*κ*B*α*/I*κ*B*α* in the articular cartilage of OA rats treated with or without acupuncture were analyzed by western blot, with GAPHD serving as a reference gene. ^ ^ *P* < 0.01, ^*∗∗∗*^*P* or ^ ^ ^ *P* < 0.001; ^*∗*^ vs. Sham; ^ vs. Model (OA: osteoarthritis; MMP-9: matrix metallopeptidase-9; SIRT1: NAD-dependent deacetylase sirtuin-1; ADAMTS5: a disintegrin and metalloproteinase with thrombospondin motifs 5).

A previous study has documented that overexpression of SIRT1 reversed IL-1*β*-induced upregulation of osteoarthritic genes including MMP-9 and ADAMTS5 in human chondrocytes [21]. By western blot, we found that SIRT1 expression level in rat articular cartilages was lower in the Model group than that in the Sham group (*P* < 0.001), while MMP-9 and ADAMTS5 expression levels of rat articular cartilages in the Model group exceeded those in the Sham group (*P* < 0.01, *P* < 0.001; Figure 2(a) and 2(b)). Treatment with acupuncture reversed those OA-associated downregulation of SIRT1 and upregulations of MMP-9 and ADAMTS-5 (*P* < 0.01, *P* < 0.001; Figures 2(a) and 2(b)).

Additionally, suppressed NF-*κ*B signaling pathway is recognized as a mechanism for mechanical stress-induced protection against OA [24]. Western blot analysis verified that the expressions of p-p65/p65 and p-I*κ*B*α*/I*κ*B*α* were increased in OA rat articular cartilages, while those increases were reversed by treatment with acupuncture (*P* < 0.01, *P* < 0.001; Figures 2(c) and 2(d)).

### 3.2. Acupuncture Inhibited OA-Associated Inflammation, the NF-*κ*B Signaling Pathway, and ECM Degradation through Upregulating SIRT1 Expression in Rat Articular Cartilages

**Figure 3:**
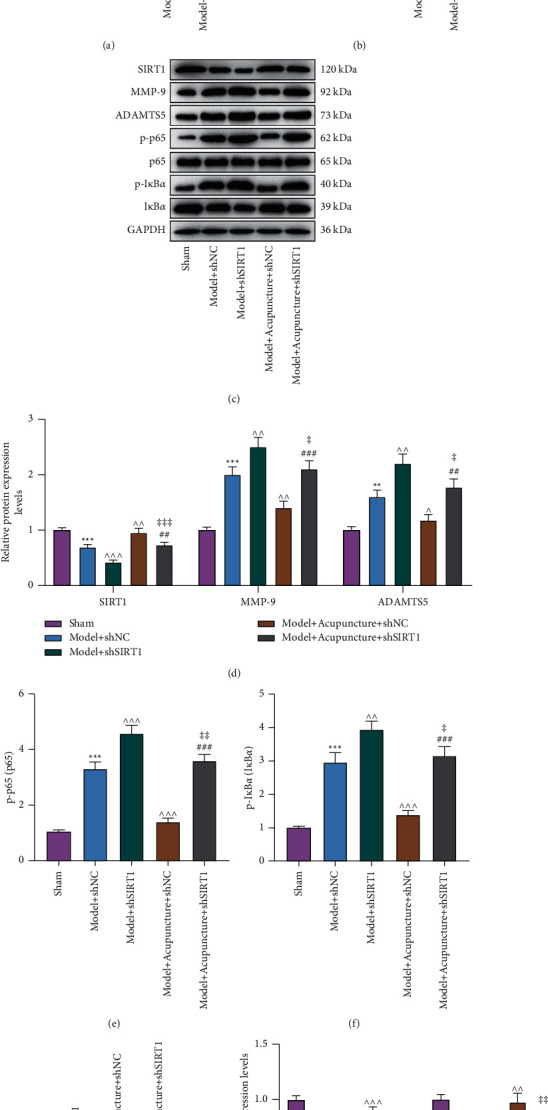
Acupuncture inhibited OA-associated inflammation, the NF-*κ*B signaling pathway, and ECM degradation through upregulating SIRT1 expression in rat articular cartilages. (a/b): The levels of TNF-*α* and IL-2 in the serum of acupuncture-treated OA rats injected with or without shSIRT1 lentiviruses were assessed by enzyme-linked immunosorbent assay. (c/d/e/f/g/h): The expressions of SIRT1, MMP-9, ADAMTS5, p-p65/p65, p-I*κ*B*α*/I*κ*B*α*, collagen II, and aggrecan in the articular cartilage of acupuncture-treated OA rats injected with or without shSIRT1 lentiviruses were analyzed by western blot, with GAPHD serving as a reference gene. ^∧^*P*or ^‡^*P* < 0.05; ^*∗∗*^*P* or ^∧∧^*P* or ^##^*P* or ^‡‡^*P* < 0.01; ^*∗∗∗*^*P* or ^^^*P* or ^###^*P* or ^‡‡‡^*P* < 0.001; ^*∗*^ vs. Sham; ∧ vs. Model + shNC; # vs. Model + Acupuncture + shNC; ‡ vs. Model + shSIRT1 (OA: Osteoarthritis; TNF-*α*: Tumor necrosis factor-*α*; IL-2: interleukin-2; shNC: shRNA-negative control; MMP-9: matrix metallopeptidase-9; SIRT1: NAD-dependent deacetylase sirtuin-1; ADAMTS5: a disintegrin and metalloproteinase with thrombospondin motifs 5).

To investigate SIRT1-associated influences in OA-related gene expressions, we injected lentivirus vectors inserted with shSIRT1 into the affected articular cavity of OA rats. Through ELISA, evident upregulations of TNF-*α* and IL-2 were detected in the Model + shSIRT1 group (*P* < 0.001; Figures 3(a) and 3(b)). Also, western blot proved that MMP-9 and ADAMTS5 levels were augmented, and SIRT1 level was dwindled in the Model + shSIRT1 group (*P* < 0.001; Figures 3(c)–3(d)). Moreover, the expressions of p-p65/p65 and p-I*κ*B*α*/I*κ*B*α* were all upregulated in the Model + shSIRT1 group (*P* < 0.001; Figures 3(c), 3(e), and 3(f)). All the above shSIRT1-induced expression changes in OA rats could be reversed by treatment with acupuncture (*P* < 0.05, *P* < 0.01, *P* < 0.001; Figures 3(a)–3(f)), but the effects of acupuncture were reversed by shSIRT1 (*P* < 0.01, *P* < 0.001; Figures 3(a)–3(f)).

Furthermore, collagen II and aggrecan in the main proteins constitute ECM which surrounds chondrocytes in cartilages [25]. It was reflected by western blot that OA rat articular cartilages exhibited downregulations of collagen II and aggrecan (*P* < 0.01, *P* < 0.001), which was further aggravated by shSIRT1 injection (*P* < 0.05). In contrast, treatment with acupuncture reversed the above inhibitory effects of OA (*P* < 0.01, *P* < 0.001) and shSIRT1 (*P* < 0.05, *P* < 0.01; Figures 3(g) and 3(h)). Certainly, shSIRT1 injection could abolish acupuncture treatment-induced reversal in the downregulations of collagen II and aggrecan in OA rats (*P* < 0.01, *P* < 0.001; Figures 3(g) and 3(h)).

## 4. Discussion

OA induces pain, restricts movement, and even disability, significantly affecting the living quality of the patients [26]. Cartilage degeneration as a result of enhanced chondrocyte catabolism or apoptosis and degraded ECM can cause friction between the bones to further trigger OA [3]. In this pathogenic process, those dysregulated ECM and chondrocytes, referred to as destroyed cartilage homeostasis, can be stimulated by inflammation [27, 28]. Therefore, a treatment method, being capable of effectively and long-lastingly resisting inflammation as well as maintaining cartilage homeostasis, might well alleviate AO pathology.

Emerging evidence has indicated that acupuncture provides certain therapeutic effects encompassing downregulating effective proinflammatory cytokine levels to improve function and relieve pain in patients with knee OA [29, 30]. Soluble proinflammatory cytokines such as IL-1*β*, TNF-*α*, and IL-6 are found to be secreted from OA cartilages via paracrine or autocrine mechanisms [28]. Elevated levels of TNF-*α* and Th1 cell-secreted cytokine IL-2 are also detected in the serum of OA rats [31]. Among these cytokines, TNF-*α*, in particular, exerts a regulatory effect on ECM degradation [32]. Downregulated synthesis of major ECM components can result from inhibited chondrocyte anabolic activities caused by TNF-*α* [32], which makes TNF-*α* a prime target in the treatment of OA. IL-2, which has a central role in the negative mediation of the immune system, is also a proinflammatory cytokine that contributes to cartilage damage [33]. Consistent with the previous finding [31], our study confirmed that OA rats exhibited upregulations of TNF-*α* and IL-2 in the serum and reaffirmed the acupuncture-induced anti-inflammatory effects as mentioned in emerging evidence [29, 30] based on reduced levels of these cytokines after acupuncture treatment. Moreover, through safranin-O staining, we observed morphological changes including cartilage erosion and loss of proteoglycan and cells in OA rat articular cartilages, which were in line with the morphological changes of cartilages in previous studies [24, 34]. Meanwhile, the TUNEL assay in our study verified that chondrocyte apoptosis was enhanced in OA rat articular cartilages, which was consistent with previous findings where chondrocyte apoptosis was closely associated with cartilage destruction [35]. After acupuncture was performed, we observed alleviated morphological changes and less apoptotic chondrocytes, which implied that acupuncture can be conducive to sustaining the structure of cartilage by inhibiting chondrocyte apoptosis.

A previous study has demonstrated that inhibited SIRT1 expression leads to facilitated progression of OA as well as enhanced chondrocyte apoptosis [36]. SIRT1 can crucially participate in cellular processes including cell apoptosis, senescence, and inflammation [37, 38]. Overexpression of SIRT1 inhibits IL-*β*-induced upregulations of ADAMTS-5 and MMP-1, 2, 9, and 13 in chondrocytes [21]. Therein, MMPs are key enzymes contributing to ECM degradation and further cartilage degeneration during OA pathogenesis [39]. MMP-9, which is increasingly expressed in arthritis, degrades the noncollagen matrix components of the cartilage [39]. Also, ADAMTS5 is a pivotal proteinase responsible for aggrecan depletion, a process destroying the collagen network in the cartilage [40]. Aggrecan, as the proteoglycan molecule that constructs ECM, can be degraded by the major collagenase MMP-13 during OA pathogenesis [39]. Not surprisingly, low-expressed SIRT1 can downregulate the aggrecan level and upregulate the ADAMTS5 level to promote OA progression [41]. Besides, collagen II, another main cartilage component relatively specific for hyaline cartilage, is lost in OA pathology [42]. Our study demonstrated that in the rat articular cartilage, OA-induced downregulations of SIRT1, collagen II, and aggrecan and upregulations of MMP-9 and ADAMTS5 were all reversed by the acupuncture treatment, manifesting that acupuncture provides certain protective effects on the structure of the cartilage.

In addition, the NF-*κ*B signaling pathway is an essential inflammatory mediator widely involved in the inflammatory responses in OA pathology [3]. NF-*κ*B-dependent signaling pathways can be potentiated by matrix degradation products, which thereby promote the secretion of proinflammatory cytokines and further accelerates cartilage degeneration [3]. Moreover, NF-*κ*B can coordinate abnormal cartilage catabolic pathways by directly or indirectly inducing catabolic gene expression such as MMP-9 and ADAMTS5 [43,44]. NF-*κ*B consists of homo- and hetero-dimers of five members of the Rel family, which includes p65 [45]. When the NF-*κ*B signaling is stimulated, p65, which is retained via interaction with low-expressed I*κ*B proteins in the cytoplasm under basal status, is phosphorylated after the phosphorylation of I*κ*B [3]. Our study detected the activation of the NF-*κ*B signaling pathway, as evidenced by increased ratios of p-p65 to p65 and p-I*κ*B*α* to I*κ*B*α* in OA rat articular cartilages. However, this activation was reversed by acupuncture treatment, connoting that acupuncture hinders the NF-*κ*B signaling pathway to suppress cartilage degeneration in OA.

Eventually, we silenced SIRT1 to fathom out whether SIRT1 plays a dominant role in the abovementioned inflammation and cartilage matrix degradation. Consequently, we unveiled that SIRT1 silencing promoted the secretion of proinflammatory cytokines and cartilage-degrading enzymes and lessened the contents of cartilage-constituting proteins, which indicates that maintaining SIRT1 expression is essential to cartilage sustainment in OA pathology.

## 5. Conclusion

In conclusion, this study authenticates that acupuncture treatment can delay OA-associated cartilage degeneration through upregulating SIRT1 expression to inhibit chondrocyte apoptosis, inflammation, NF-*κ*B signaling activation, and cartilage matrix degradation.

## Data Availability

The analyzed datasets generated during the study are available from the corresponding author on reasonable request.
